# BolA Is Required for the Accurate Regulation of c-di-GMP, a Central Player in Biofilm Formation

**DOI:** 10.1128/mBio.00443-17

**Published:** 2017-09-19

**Authors:** Ricardo N. Moreira, Clémentine Dressaire, Susana Barahona, Lisete Galego, Volkhard Kaever, Urs Jenal, Cecília M. Arraiano

**Affiliations:** a Instituto de Tecnologia Química e Biológica António Xavier, Universidade Nova de Lisboa, Oeiras, Portugal; b Research Core Unit Metabolomics and Institute of Pharmacology, Hannover Medical School, Hannover, Germany; c Biozentrum of the University of Basel, Basel, Switzerland; National Cancer Institute

**Keywords:** biofilm, BolA, diguanylate cyclase, motility, phosphodiesterase, c-di-GMP

## Abstract

The bacterial second messenger cyclic dimeric GMP (c-di-GMP) is a nearly ubiquitous intracellular signaling molecule involved in the transition from the motile to the sessile/biofilm state in bacteria. C-di-GMP regulates various cellular processes, including biofilm formation, motility, and virulence. BolA is a transcription factor that promotes survival in different stresses and is also involved in biofilm formation. Both BolA and c-di-GMP participate in the regulation of motility mechanisms leading to similar phenotypes. Here, we establish the importance of the balance between these two factors for accurate regulation of the transition between the planktonic and sessile lifestyles. This balance is achieved by negative-feedback regulation of BolA and c-di-GMP. BolA not only contributes directly to the motility of bacteria but also regulates the expression of diguanylate cyclases and phosphodiesterases. This expression modulation influences the synthesis and degradation of c-di-GMP, while this signaling metabolite has a negative influence in *bolA* mRNA transcription. Finally, we present evidence of the dominant role of BolA in biofilm, showing that, even in the presence of elevated c-di-GMP levels, biofilm formation is reduced in the absence of BolA. C-di-GMP is one of the most important bacterial second messengers involved in several cellular processes, including virulence, cell cycle regulation, biofilm formation, and flagellar synthesis. In this study, we unravelled a direct connection between the *bolA* morphogene and the c-di-GMP signaling molecule. We show the important cross-talk that occurs between these two molecular regulators during the transition between the motile/planktonic and adhesive/sessile lifestyles in *Escherichia coli*. This work provides important clues that can be helpful in the development of new strategies, and the results can be applied to other organisms with relevance for human health.

## IMPORTANCE

Bacterial cells have evolved several mechanisms to cope with environmental stresses. BolA-like proteins are widely conserved from prokaryotes to eukaryotes, and in *Escherichia coli*, in addition to its pleiotropic effects, this protein plays a determinant role in bacterial motility and biofilm formation regulation. Similarly, the bacterial second messenger c-di-GMP is a molecule with high importance in coordinating the switch between planktonic and sessile life in bacteria. Here we have unravelled the importance of accurate regulation of cross-talk between BolA and c-di-GMP for a proper response in the regulation of these bacterial lifestyles. This finding underlines the complexity of bacterial cell regulation, revealing the existence of one additional tool for fine-tuning such important cellular molecular mechanisms. The relationship between BolA and c-di-GMP gives new perspectives regarding biofilm formation and opens the possibility to extend our studies to other organisms with relevance for human health.

## INTRODUCTION

The ability of bacteria to sense and adapt to environmental changes is critical for survival. Under stress conditions, prokaryotic cells rapidly adjust their gene expression in order to induce the physiological and molecular adaptations needed. The *Escherichia coli* (*E. coli*) *bolA* gene is induced at the onset of stationary phase and in response to several stresses, leading to substantial changes in the cell (1, 2). BolA expression is tightly regulated at the transcriptional and post-transcriptional levels (1, 3–5). Under optimal growth conditions, *bolA* transcription is regulated by a constitutive promoter, *bolAp2*, whose activity depends on the housekeeping sigma factor σ^70^ (1). Under harsh environmental conditions, its expression is mostly driven by a *gearbox* promoter, *bolAp1*, controlled by sigma factor σ^S^ (1, 2, 6). Additionally, *bolA* expression is repressed by the histone-like protein H-NS (7) and by OmpR in its phosphorylated form (8). The post-transcriptional regulation of *bolA* mRNA levels involves both RNase III and poly(A) polymerase I (PAPI) (5, 9).

BolA and its homologues constitute a protein family that is widely conserved across prokaryotes and eukaryotes (10). Functional studies have associated BolA with a range of cellular processes, such as bacterial morphology, membrane permeability, motility, and biofilm formation (reviewed in reference 11). However, the biological details regarding its mechanism of action in the regulation of biofilm were only recently unravelled (12). BolA was shown to repress flagellar synthesis and induce TCA cycle-related genes, with consequences for bacterial motility (12).

One of the most extensively studied factors involved in the transition from the motile state to the non-motile/biofilm state in bacteria is the bacterial second messenger c-di-GMP (13). This molecule is synthesized by diguanylate cyclases (DGCs), whose activity has been associated with the highly conserved GGDEF protein domain (14). On the other hand, its hydrolysis is carried out by phosphodiesterases (PDEs), enzymes with an EAL or HD-GYP domain (15, 16). C-di-GMP was shown to be involved in several cellular processes, including cell differentiation (17, 18), cell cycle progression (19), biofilm formation and dispersal (20–22), and cell motility (13, 23). One of the most remarkable features of c-di-GMP is its ability to regulate the transition from the planktonic lifestyle to the sessile lifestyle (13, 24). The increased production of c-di-GMP by certain DGCs has a negative effect on cell motility and strongly activates the production of adhesins and biofilm matrix components (13). In contrast, low levels of c-di-GMP, associated with the activity of PDEs, promote motility and increase bacterial dispersion (23, 25). Thus, both BolA and c-di-GMP play key roles in the transition between the planktonic and the sessile lifestyles, repressing cell motility and promoting biofilm development (12, 26).

In this report, we have unravelled a direct connection between the *bolA* morphogene and the signaling molecule c-di-GMP. We show the important cross-talk that occurs between these two molecular regulators during the transition between the motile/planktonic and adhesive/sessile lifestyles in *E. coli*.

## RESULTS

### BolA controls cellular levels of c-di-GMP.

**FIG 1:**
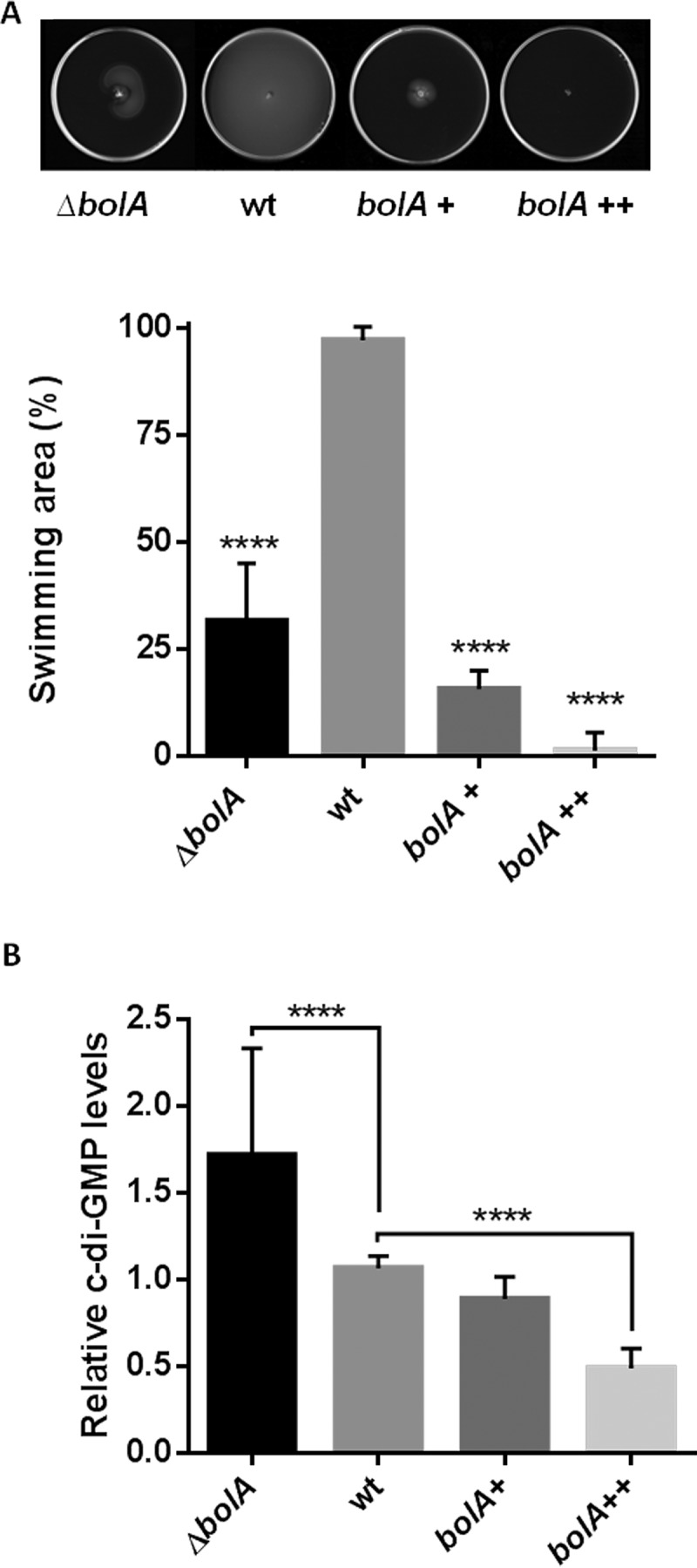
Influence of BolA in cell motility and c-di-GMP regulation. (A) To measure motility, bacteria were inoculated in swimming agar plates. The plates were incubated at 37°C for 17 h and pictures were taken using ImageScanner III (GE Healthcare Life Sciences). Significant differences, relative to the wt strain, were determined by measuring the diameter of the swimming halo. ****, *P* < 0,0001 (analysis of variance [ANOVA] test). (B) Quantification of c-di-GMP metabolite in bacteria expressing different amounts of BolA. In the absence of BolA, the c-di-GMP concentration in the cells was increased about 1.7-fold. In the presence of high levels of this protein, the c-di-GMP concentration was reduced. ****, *P* ≤ 0.0001 (Student’s *t* test).

The absence of BolA was reported to affect the ability of *E. coli* to develop biofilms (27). Mutants lacking *bolA* showed reduced biofilm formation and *bolA* overexpression led to a significant increase in bacterial aggregation (27). Bacterial motility is particularly important at early stages of surface colonization in *E. coli* (28). To evaluate the role of BolA in *E. coli* motility, the swimming capacity of the bacterium was tested in the wild-type (wt) strain and compared with the swimming capacity of *bolA* deletion (Δ*bolA*) mutant and *bolA*-overexpressing strains. The overexpression was obtained with a plasmid expressing *bolA* either under the control of its own promoters (*bolA*^+^) or under the control of an arabinose-inducible promoter (*bolA*^++^). In Fig. 1A, it is possible to observe the differences between the strains expressing different quantities of BolA. In the BolA-overproducing strains, motility was reduced, in agreement with what was previously shown (12). Since the ability to form biofilm is partially impaired in the *bolA* deletion mutant (27), it was expected that these bacteria would swim more efficiently than the wt strain. Surprisingly, the motility of the *bolA* mutant was also reduced. Thus, both the absence of and overexpression of *bolA* compromise the ability of *E. coli* cells to spread on semisolid agar plates.

In bacteria, YcgR reduces motility by interacting directly with flagellar motor proteins, slowing down flagellar rotor speed and altering the frequency of the rotational switch (29–31). The motility impairment obtained in the Δ*bolA* strain could be associated with an upregulation of YcgR. Therefore, we have analyzed the influence of BolA absence in regard to YcgR expression. According to the transcriptomic data, variations at the mRNA of *ycgR* were not detected (data not shown). Despite this fact, a *bolA ycgR* double mutant was constructed and the swimming ability of *E. coli* was analyzed. If the effect on swimming occurred via YcgR, even by an indirect pathway, we would expect to observe a reversion of the Δ*bolA* strain phenotype. However, our results were inconclusive and it was not possible to clarify the influence of BolA in the YcgR-dependent flagellar rotation mechanism.

The ability of bacteria to regulate the planktonic-to-sessile lifestyle transition is also well known to involve the bacterial second messenger c-di-GMP (13, 25). This molecule is involved in positive regulation of curli synthesis with a negative impact in flagellar biogenesis cascade (32) and has been described as an important factor in the transition between the motile and non-motile lifestyles in bacteria (13). Since both BolA and c-di-GMP seem to be regulating the motile/sessile transition, leading to similar phenotypes, we were interested in analyzing whether the presence or absence of BolA had any impact in the regulation of c-di-GMP intracellular levels. Different samples, representative of the different bacterial growth stages, were processed for c-di-GMP quantification by liquid chromatography coupled with tandem mass spectrometry (Fig. 1B). The levels of this metabolite were compared in the four different strains. In the exponential and late exponential phases of growth, the amount of c-di-GMP was below the detection level and thus could not be reproducibly quantified (data not shown). However, in the early stationary phase, the levels of c-di-GMP were significantly more nearly constant and were detectable in all extracts corresponding to the different strains (Fig. 1B). When *bolA* was overexpressed using the plasmid with its own promoters (*bolA*^+^), no difference in c-di-GMP levels in comparison to the wt strain levels was observed. However, the *bolA*^++^ strain showed a decrease in c-di-GMP levels of about 2-fold in comparison to the wt strain. Finally, in the *bolA* deletion mutant, the c-di-GMP levels in the cells increased 2-fold relative to the levels seen with the wt strain. This observation supports the data corresponding to the motility impairment previously detected in this strain; furthermore, it links BolA with the regulation of the c-di-GMP metabolite in bacteria for the first time.

### BolA is involved in the transcriptional regulation of diguanylate cyclases and phosphodiesterases.

**FIG 2:**
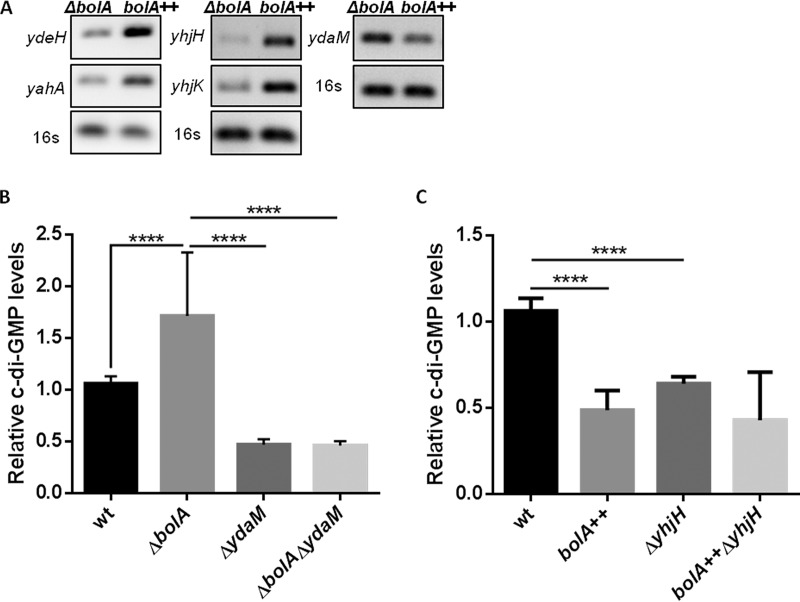
Validation of microarrays results by RT-PCR and analysis of BolA effect on the intracellular c-di-GMP levels in *ydaM* and *yhjH* knockout mutants. (A) The levels of several DGCs and PDEs differentially regulated by BolA were determined to validate the data from the microarrays. In all reactions, 50 ng of total RNA that had been extracted 3 h after the addition of arabinose to the media was used. The results of parallel RT-PCR procedures run in the absence of reverse transcriptase yielded no product. The results of RT-PCR performed with primers specific for 16S rRNA showed that there were no significant variations in the amount of RNA used in each sample. The images are representative of results from at least three independent RT-PCR experiments performed with RNAs from two different extractions. (B and C) A *bolA ydaM* double mutant showed a reversion effect on the elevated levels of c-di-GMP caused by a single *bolA* mutation (B). In the *bolA*^++^ strain lacking the *yhjH* gene, inhibition of c-di-GMP production was retained (C). Both observations indicate a modulation of c-di-GMP levels dependent on BolA through the regulation of *ydaM* and *yhjH* gene expression. ****, *P* ≤ 0.0001 (one-way ANOVA test).

The results presented in Fig. 1 indicate that BolA impacts the intracellular levels of c-di-GMP. In order to further estimate the effect of BolA protein in such a mechanism, we were interested in studying the role of this protein in c-di-GMP synthesis and degradation. The effects of BolA in the global transcription profile of *E. coli* were previously studied (12). These transcriptomic results allowed us to identify several enzymes responsible for the synthesis (DGCs) and degradation (PDEs) of c-di-GMP that were differentially regulated in the presence of elevated levels of BolA. The fold change data and associated statistical significance can be observed in Table 1. Representatives of both groups of enzymes were found among the targets that were differentially regulated. The results from the transcriptome analysis (Δ*bolA* strain versus *bolA*^++^ strain) were validated by (semi)quantitative reverse transcription PCR (RT-PCR) (Fig. 2A). All genes were confirmed to vary in similar ways with respect to the transcriptome data, with the single exception of *yhjK*. Of the total number of DGCs of *E. coli*, seven were regulated in a BolA-dependent manner. Among the identified targets, the levels of YdaM, one of the best-characterized DGCs, were reduced about 30% in the presence of BolA. Regarding the genes encoding PDEs, five were differentially regulated by BolA, with three of them (*ydiV*, *yliE*, and *yahA*) presenting a difference representing an increase of more than 3.3-fold in the mRNA level in comparison to the Δ*bolA* strain results. Additionally, *yhjH*, encoding the PDE involved in the regulation of the mechanism of flagellar motor rotation (33, 34), was among the targets regulated. This phosphodiesterase acts antagonistically to YegE and YedQ DGCs, having a major effect on cell motility (29, 35). On the other hand, YdaM, by acting together with YciR and MlrA, is described as the most important protein involved in *csgD* transcription, an extremely important player in biofilm formation. Importantly, these enzyme modules act as a sequential pathway in which regulation of c-di-GMP levels is a determinant required to achieve a correct cellular response.

**TABLE 1:** Differentially regulated genes involved in bacterial c-di-GMP synthesis and degradation^a^

Gene and enzyme category	Fold change	*P* value
Diguanylate cyclases		
* yfiN*	2.04	1.61E^−02^
* yliF*	1.97	1.13E^−02^
* yneF*	2.65	2.73E^−03^
* yhjk*	0.72	7.61E^−02^
* ydeH*	2.86	1.41E^−03^
* ycdT*	2.65	3.43E^−03^
* ydaM*	0.69	4.31E^−02^
		
Phosphodiesterases		
* ydiV*	5.58	6.70E^−04^
* yliE*	3.38	1.73E^−03^
* ycgF*	1.49	7.79E^−02^
* yhjH*	1.84	5.10E^−03^
* yahA*	3.80	2.84E^−03^

a Fold change data represent the *bolA*^++^/Δ*bolA* ratios for several genes responsible for the expression of proteins involved in the regulation of c-di-GMP. The *P* value associated with each gene represents the level of statistical significance of the differential expression.

Since the expression of two of the most important enzymes involved in the synthesis and degradation of c-di-GMP (YdaM DGC and YhjH PDE) was influenced by BolA, we were interested in evaluating whether the elevated and reduced levels of c-di-GMP in the *ΔbolA* and *bolA*^++^ strains, respectively, could be due to the differential levels of expression of these enzymes. Both proteins were previously described as participating in an important cascade of c-di-GMP regulation (36). To test this hypothesis, c-di-GMP levels were estimated in a double mutant *bolA ydaM* strain and in the *bolA*^++^ Δ*yhjH* strain (Fig. 2B and C). In fact, in the absence of *bolA* and *ydaM*, there was a significant (70%) reduction in the levels of c-di-GMP in comparison to that seen with the single Δ*bolA* mutant (Fig. 2B). Moreover, the levels of c-di-GMP under these conditions were observed to be similar to those seen with the single *ydaM* mutant, which underlines the role of BolA in regulating c-di-GMP via *ydaM*. Since this DGC was previously described as not affecting motility in bacteria (35), as expected, changes were not observed in the swimming phenotype of the Δ*bolA* mutant when *ydaM* was simultaneously absent (see Fig. S1A in the supplemental material). Regarding the *bolA*^++^ Δ*yhjH* strain, surprisingly, it did not show an increase in the level of intracellular c-di-GMP (Fig. 2C). However, a single mutation of *yhjH* originated a similar phenotype. This promptly showed us that even though BolA was binding to *yhjH* promoters and affecting its RNA levels, it was not increasing c-di-GMP levels exclusively through this pathway. The absence of this PDE is described to impair the swimming of bacteria (37). Given this, testing the swimming phenotype of the *bolA*^++^ Δ*yhjH* double mutant, cumulative effects on motility were observable (Fig. S1B).

10.1128/mBio.00443-17.1 FIG S1  Analysis of BolA effect on the motility of a *ydaM yhjH* knockout mutant. (A) The *bolA ydaM* double mutant showed a phenotype similar to that of the *bolA* single mutant. (B) In the *bolA*^++^ strain lacking the *yhjH* gene, due to the high levels of BolA that were present simultaneously with the increased levels of c-di-GMP, a cumulative effect on motility impairment was observed. All plates were incubated at 37°C, and photographs were taken using ImageScanner III (GE Healthcare Life Sciences). Statistically significant differences were determined by measuring the diameter of the swimming halo. ****, *P* < 0.0001 (one-way ANOVA test). Download FIG S1, TIF file, 1.3 MB. Copyright © 2017 Moreira et al. 2017 Moreira et al. This content is distributed under the terms of the Creative Commons Attribution 4.0 International license.

To further study the biological significance of the differential levels of expression of these enzymes, biofilm formation was tested (Fig. S2). As a control, the wt, Δ*bolA*, and *bolA*^++^ strains were compared and verified to have phenotypes similar to those described in the literature. A knockout (Δ*bolA*) mutant was previously shown to be impaired in biofilm formation in comparison to a wt strain (27). Together with the results obtained so far, this could have been related to the 1.7-fold increase in c-di-GMP levels in this strain. To provide evidence for this, a comparison between the biofilm formation levels of the Δ*bolA* and Δ*bolA* Δ*ydaM* mutants was done. In fact, despite the reduction in c-di-GMP levels in the Δ*bolA* Δ*ydaM* mutant, this strain showed a similar quantity of biofilm biomass, nearly matching the wt condition. Finally, regarding the *yhjH* mutant, as can be observed in Fig. S2, the levels of biofilm matched the levels seen with the *bolA*^++^ strain. Given this, we believe that the effect of BolA overexpression is dominant with respect to biofilm formation due to its strong positive participation in the regulation of the curli/fimbriae synthesis pathway (12). Together, these observations indicate the possible importance of accurate levels of c-di-GMP in the cell for proper biofilm formation development.

10.1128/mBio.00443-17.2 FIG S2  Analysis of BolA-dependent c-di-GMP regulation in biofilm formation. Biofilm biomass was estimated by the crystal violet method after 24 h of growth. **, *P* < 0.05; ****, *P* ≤ 0.0001 (one-way ANOVA test). Download FIG S2, TIF file, 1.2 MB. Copyright © 2017 Moreira et al. 2017 Moreira et al. This content is distributed under the terms of the Creative Commons Attribution 4.0 International license.

In conclusion, BolA not only contributes directly to the motility of bacteria (12) but also participates in the expression of DGCs and PDEs, influencing the synthesis and degradation of the secondary signaling metabolite c-di-GMP.

### C-di-GMP levels are regulated through the direct binding of BolA to the promoters of diguanylate cyclases and phosphodiesterases.

**FIG 3:**
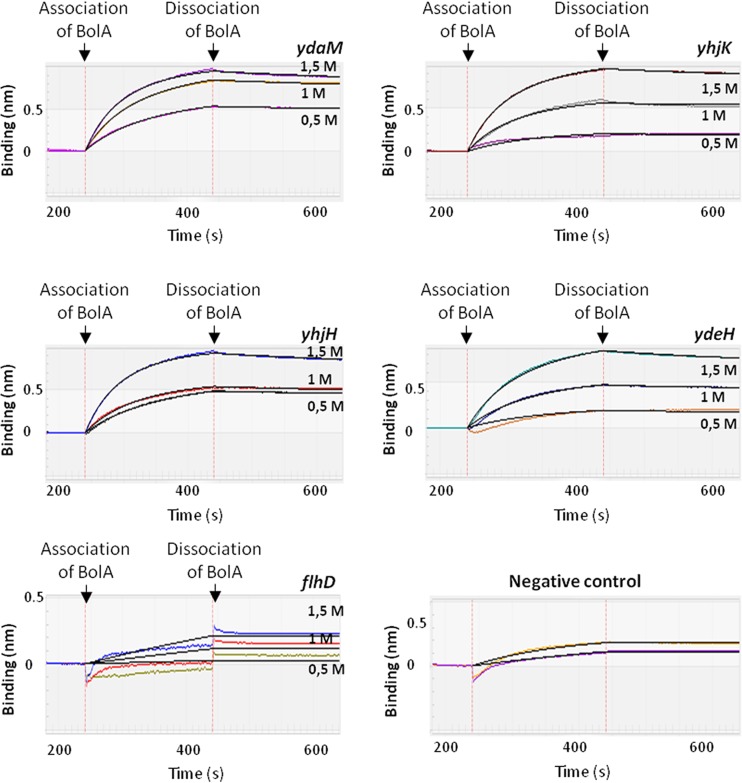
Real-time biolayer interferometry measurement of 0.5, 1.0, and 1.5 M BolA in immobilized substrate. BolA association and dissociation data are labeled and represented by the dashed red line. BSA protein and *flhDC* promoter sequence were used as negative controls.

Previous results showed the ability of *E. coli* BolA to directly interact *in vivo* and *in vitro* with DNA molecules, influencing the rate of transcription of a given gene (12, 38, 39). The data showing the differential regulation of DGCs and PDEs led us to investigate whether BolA was influencing their expression in a direct manner. For that investigation, a real-time biolayer interferometry technique was performed. Purified BolA protein was used to test the interaction with different substrates corresponding to the promoter regions of DGCs and PDEs. The DNA fragments amplified for this study were selected on the basis of statistical analyses of the results of a previously performed chromatin immunoprecipitation sequencing (ChIP-seq) experiment (12). The results of the protein-DNA interaction can be observed in Fig. 3 and Table 2. BolA was able to interact directly with all tested substrates with a dissociation constant in the nanomole range. The *ydaM*, *yhjH*, and *yhjK* promoters were all associated with a dissociation constant of about 35.0 nM, while the *ydeH* promoter presented an approximately 2-fold-higher dissociation constant (81.1 nM). Together, our data suggest that BolA plays an important role in the regulation of the enzymes responsible for the synthesis and degradation of the c-di-GMP signaling molecule.

**TABLE 2:** Affinity measurement by biolayer interferometry^a^

Gene/protein	*K*_*D*_ (M)	*K*_*a*_ (1/ms)	*K*_*a*_ error	*K*_*d*_ (1/s)	*K*_*d*_ error
*ydaM*	3.54E^−08^	1.14E^+04^	7.61E^+01^	4.03E^−04^	9.48E^−06^
*yhjH*	3.53E^−08^	1.22E^+04^	8.79E^+01^	4.29E^−04^	1.18E^−05^
*yhjK*	3.80E^−08^	1.06E^+04^	1.67E^+02^	4.01E^−04^	2.33E^−05^
*ydeH*	8.11E^−08^	7.74E^+03^	2.05E^+02^	6.27E^−04^	3.38E^−05^
*flhD*	ND	ND	ND	ND	ND
*yhjK*/BSA	ND	ND	ND	ND	ND

a Equilibrium dissociation constants (*K*_*D*_) were determined by biolayer interferometry using a BLItz system (ForteBio Inc.) according to the results of an advanced kinetics experiment. *K*_*a*_, association rate constant; *K*_*d*_, dissociation rate constant; *K*_*D*_, equilibrium dissociation constant of the reaction; ND, not determined.

### The transcription of *bolA* is c-di-GMP dependent.

Different types of c-di-GMP effector proteins with downstream consequences were previously unravelled with respect to gene regulation in the cell (reviewed in reference 40). This may occur as a consequence of the interaction of the second messenger with a transcription regulator such as FleQ (41) or VpsT (21). It is also known that there are riboswitches that bind c-di-GMP, followed by a second element that modulates gene expression at the transcriptional or translational level (42).

**FIG 4:**
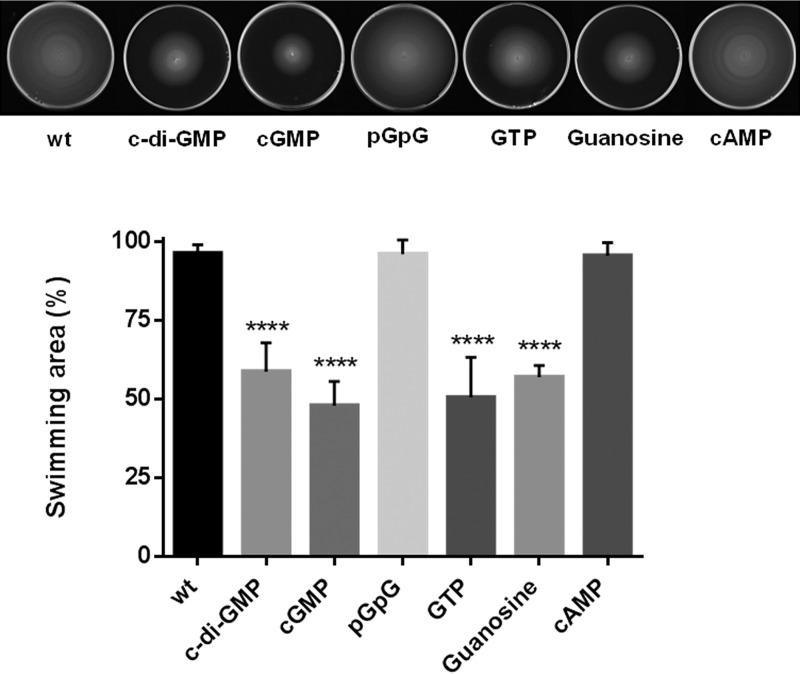
Motility assay of a wt strain growing in LB and in LB supplemented with 10 µM c-di-GMP, cGMP, GTP, pGpG, or guanosine. When growth medium was supplemented with c-di-GMP, similar effects of elevated intracellular levels of this metabolite were observed, representing inhibition of bacterial motility. The same effect was observed when the medium was supplemented with cGMP, GTP, and guanosine but not when it was supplemented with pGpG. cAMP, a metabolite that is not related to c-di-GMP, was used as negative control under the same conditions. Significant differences were determined by measuring the swimming halo diameter after 10 h of growth. ****, *P* < 0.0001 (one-way ANOVA test).

Since our discoveries indicate a possible role of BolA in the c-di-GMP balance in *E. coli*, we were interested in investigating whether this metabolite could have an influence on the transcription of the *bolA* gene. With this purpose in mind, an artificial condition where c-di-GMP would be abundant in the growth environment was created. For that, authentic c-di-GMP was added as a supplement in the growth media. Since this metabolite is known to reduce bacterial motility in semi-solid agar due to flagellum rotation impairment (29), wt *E. coli* cultures were used for a first screening. As shown in Fig. 4, when growth medium was supplemented with 10 µM c-di-GMP, the bacterial swimming capacity was significantly reduced. Moreover, this was also verified with the addition of the related signaling molecule cGMP, with guanosine, and with the precursor nucleotide GTP. However, c-di-GMP direct degradation product pGpG did not show any effect on the swimming pattern. Together, these results may indicate not only that c-di-GMP can act via a sensor/receptor of bacteria but also that its precursors may influence intracellular signaling mechanisms.

**FIG 5:**
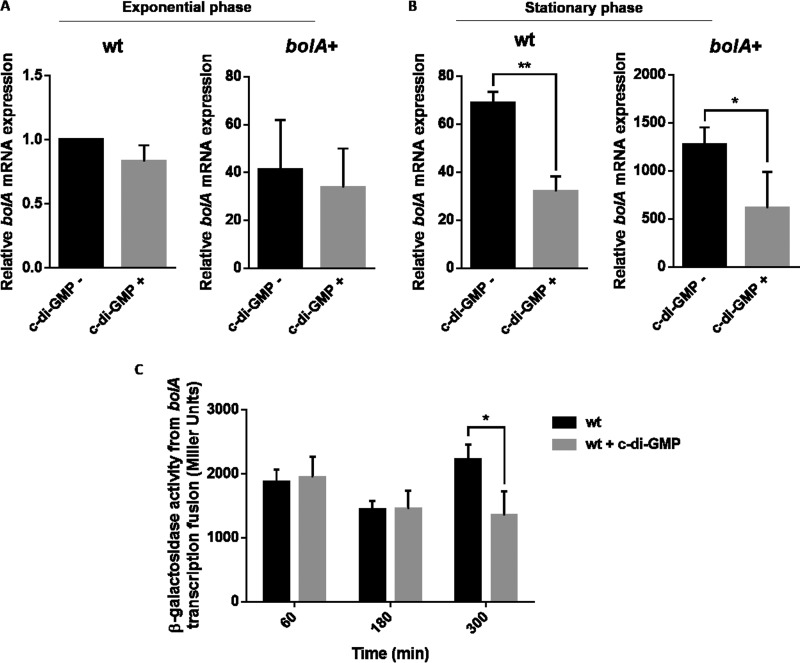
Influence of c-di-GMP in the transcription of *bolA*. (A and B) *bolA* mRNA relative expression levels determined by qRT-PCR. Samples in the exponential (A) and stationary (B) phases of growth were analyzed. The levels of expression of *bolA* were compared in wt and *bolA*^+^ strains in the presence or absence of c-di-GMP. All values are relative to expression of the wt strain in the exponential phase. (C) Graph showing the β-galactosidase activity of a construct with the *bolA* promoter region. The expression of β-galactosidase was analyzed in the wt strain cultured in LB media or in LB media supplemented with 1 µM c-di-GMP. *, *P* ≤ 0.05; **, *P* ≤ 0.01 (Student’s *t* test).

As previously mentioned, *bolA* expression is driven by two different promoters, one regulated by σ^70^ and the second regulated by σ^s^. In stationary phase, *bolA* expression is mainly controlled by the σ^s^-associated promoter, increasing the expression of *bolA* by around 70-fold (Fig. 5). Given this, wt and *bolA*^+^ strains were grown to the exponential and stationary phases in the presence or absence of c-di-GMP, and total RNA was extracted. The levels of *bolA* mRNA were analyzed by quantitative real time PCR (qRT-PCR) (Fig. 5A and B). In exponential-phase samples (Fig. 5A), there were no significant differences between the strains growing in the absence or presence of c-di-GMP in the medium. This was observed not only in the wt strain but also in the one overexpressing *bolA* under the regulation of its own promoters (strain *bolA*^+^). However, when bacteria were in the stationary phase (Fig. 5B), there was a significant reduction of about 60% in *bolA* mRNA levels in both the wt and *bolA*^+^ strains. This indicates that, independently of the presence of *bolA* gene copies in the cell, the regulation by c-di-GMP is preserved in a similar way. This regulation was additionally confirmed by transcriptional fusion performed with a reporter gene. We have cloned *bolA* promoters in a plasmid encoding the β-galactosidase enzyme. In the presence of c-di-GMP in the medium and in stationary phase, the β-galactosidase activity reduced significantly (about 40%) (Fig. 5C). This shows that there was a reduction in the transcription of *bolA* promoters dependent on c-di-GMP.

**FIG 6:**
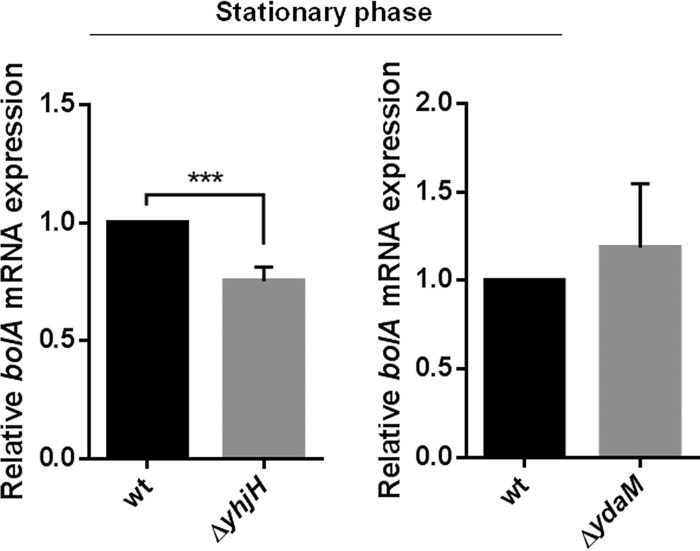
Influence of YdaM and YhjH in the transcription of *bolA*. (Left) In a mutant strain with a deletion of *yhjH* (Δ*yhjH* mutant) and consequently with higher intracellular levels of c-di-GMP, *bolA* expression was reduced. (Right) When *ydaM* was deleted, with the concomitant decrease of c-di-GMP levels, expression of *bolA* mRNA was slightly increased in comparison to the wt strain levels. ***, *P* ≤ 0.001 (Student’s *t* test).

Since the mechanism by which c-di-GMP acts externally in the intracellular metabolism has not yet been clarified, experiments employing a similar approach were performed using knockout strains for two enzymes (YhjH and YdaM) that are BolA dependent and that have been reported to be important in c-di-GMP regulation. As c-di-GMP is highly produced only during the transition from the exponential phase to the stationary phase, we performed the qRT-PCR analyses only in the stationary-phase cells for this set of samples. Similarly to what was observed before, in the absence of *yhjH* and, consequently, with the increase of c-di-GMP levels, expression of *bolA* was reduced (Fig. 6). Even if not on the same order of magnitude, it was possible to see a significant reduction of about 25% of *bolA* transcript levels. On the other hand, when *ydaM* was deleted and c-di-GMP levels were reduced, the impact in *bolA* mRNA levels was not statistically significant, even though there was still an increase of 20% in the *bolA* transcript level (Fig. 6).

Together, our results suggest not only that BolA is modulating the levels of c-di-GMP in *E. coli* by regulating the expression of DGCs and PDEs but also that the c-di-GMP signaling molecule is involved in the control of *bolA* expression. This cross-regulation seems to indicate that bacteria need to balance the amounts of these two components in order to react properly under stress conditions.

## DISCUSSION

The molecular mechanism behind the bacterial transition from the planktonic lifestyle to the biofilm lifestyle has been a fascinating object of study. Despite significant advances in the topic, there are still several interconnected pathways that need to be clarified. Here, we are adding an important piece to the puzzle by showing that BolA can interfere with this transition by balancing the intracellular concentration of the bacterial second messenger c-di-GMP.

Recently, we showed that BolA is a very important transcriptional switch, that is specifically involved in the transition between the planktonic stage and the attachment stage of biofilm formation processes (12). Elevated levels of BolA were shown to affect negatively the swimming of bacteria in semi-solid media (12). This was due to impaired flagellar assembly in a strain overexpressing BolA. Taking this into account and in agreement with the role of BolA in biofilm formation (27), it would be expected that bacteria would have improved swimming capacity in the absence of BolA. However, to our surprise, motility was impaired in the Δ*bolA* strain.

The ability of bacteria to regulate the planktonic-to-sessile lifestyle transition is well known to involve the bacterial second messenger c-di-GMP (13, 25). Its synthesis and degradation have received significant attention in recent years (43). The regulatory network of c-di-GMP is complex, partly due to the large number of enzymes which synthesize (DGCs) or degrade (PDEs) this molecule and which are encoded in the genome. In several previous studies (12, 27), BolA protein was referred to as an important bacterial protein that stimulates biofilm formation. The fact that BolA and c-di-GMP have similar functions with respect to the different stages of the bacterial life cycle (12, 25) and the fact that BolA represses flagellar synthesis, enhancing the curli biosynthetic pathway, led us to investigate if its expression had consequences for the regulation of c-di-GMP levels. Quantitative analyses of intracellular c-di-GMP revealed an increase in the concentration of this metabolite in Δ*bolA* cells. In fact, the reduced swimming seen with this strain might be associated with the 1.7-fold-increased levels of c-di-GMP.

As mentioned above, there are large numbers of DGCs and PDEs encoded in the bacterial genomes. The balance between synthesis and degradation of c-di-GMP comes from a complex network of regulation that involves both types of enzymes (23). BolA was shown to directly interact with nucleic acids in order to activate or repress gene expression (12, 38, 39). In agreement, our results have revealed that BolA can play an active role in the regulation of c-di-GMP. Transcriptomic analyses, together with biolayer interferometry, allowed us to identify the BolA targets with regard to DGCs and PDEs. Among the differentially regulated genes, there are several that encode proteins still classified as putative DGCs or PDEs. Moreover, two of the most important enzymes involved in the synthesis and degradation of c-di-GMP are transcriptionally controlled by BolA. Those are the *ydaM* DGC and *yhjH* PDE. Both genes encode proteins that have been previously described as participating in the cascade of c-di-GMP regulation linked by the YciR trigger enzyme (36). YhjH is coregulated with flagellar genes and its levels reduce with the concomitant increase of c-di-GMP levels (35). At the same time, c-di-GMP impairs the activity of another PDE protein, YciR, in binding YdaM, which allows YdaM to generate c-di-GMP and to activate the curli biosynthetic pathway (36). Our data show downregulation of *ydaM* and upregulation of *yhjH*. Moreover, the BolA effect on c-di-GMP appeared to be fully dependent upon the presence of YdaM. BolA regulation of YhjH apparently had less influence on c-di-GMP. However, *bolA* and *yhjH* mutants showed additive effects on motility, suggesting yet other targets for BolA in the motility pathway. Thus, it is plausible to speculate about interference of BolA in the cascade referred to above, that would result in fine-tuned gradual regulation of c-di-GMP in the transition of bacteria to the sessile state.

**FIG 7:**
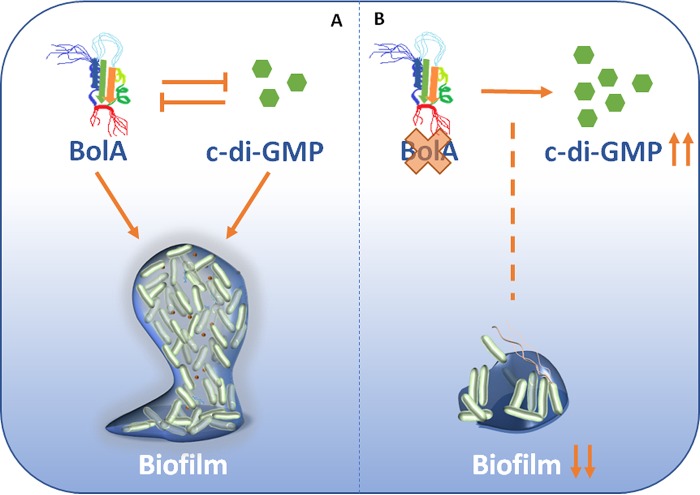
Cross-talk between the transcription factor BolA and the second messenger c-di-GMP in bacterial biofilm formation. BolA and c-di-GMP are known to be important players in biofilm development. **(**A**)** Our model suggests that a negative-feedback modulation, which leads to a balance between these two factors, is needed for a proper physiological response. **(**B**)** Additionally, the absence of BolA leads to less-robust biofilm formation, even in the presence of high levels of c-di-GMP, which evidences the determinant function of this protein in the regulation of biofilm formation.

Interestingly, the interplay between BolA and c-di-GMP is not unidirectional. *bolA* mRNA transcription is also influenced by c-di-GMP. When this molecule is present in elevated amounts in the cell, there is a reduction in *bolA* mRNA levels. This strengthens the idea of the importance of the balance between these two molecules in achieving a correct adaptation of bacteria to growth conditions. C-di-GMP is known to bind proteins containing a PilZ domain (33). As a transcription factor, BolA is able to bind nucleic acids. We believe that the balance between these two players is of major importance for tight regulation of the complex flagellar and curli synthesis pathways. High levels of c-di-GMP are strongly linked to bacterial biofilm formation (13). Taking into consideration the fact that, without BolA, c-di-GMP levels are elevated but *E. coli* biofilm formation is nevertheless partially impaired, the present work underlines the importance of BolA for a proper bacterial stress response and consequent biofilm development (Fig. 7).

In bacteria, c-di-GMP is metabolized into pGpG, which is further hydrolyzed into GMP (44). When c-di-GMP was added to the media as a supplement, its characteristic effect in bacterial swimming was observed, suggesting the existence of a mechanism by which cells can sense or internalize this second messenger molecule. Additionally, it is plausible to hypothesize that bacteria that lyse can release different molecules to the media, including enzymes that metabolize c-di-GMP. When the medium was supplemented with pGpG, no response of the bacteria to this molecule was observed. However, cGMP and the precursors GTP and guanosine had an influence on the swimming of *E. coli*. Together, these observations may indicate not only that c-di-GMP can act via a sensor/receptor of bacteria but also that the degradation product cGMP and synthesis precursors may influence intracellular signaling mechanisms. Nevertheless, the specific mechanism by which c-di-GMP regulates *bolA* expression remains to be elucidated.

Our results highlight the cross-talk that occurs between the BolA transcription factor and the second molecular messenger c-di-GMP during the transition between the planktonic and sedentary bacterial lifestyles. This finding underlines the complexity of bacterial cell regulation, revealing the existence of one additional tool for fine-tuning such an important cellular molecular mechanism. Whether the cross-talk between BolA and c-di-GMP connects to feed-forward or homeostatic regulation remains unclear. In a recent review, Caly and colleagues discussed strategies designed to control bacterial biofilm formation by targeting c-di-GMP (45). C-di-GMP is among the most important bacterial second messengers involved in many cellular processes, including differentiation, virulence, cell cycle regulation, biofilm formation, and flagellar synthesis (43). In this regard, the intricate relationship between BolA and c-di-GMP opens more options with the possibility to extend our studies to other organisms with relevance for human health.

## MATERIALS AND METHODS

### Oligonucleotides, bacterial strains, and plasmids.

All strains and plasmids used in this study are listed in Table S1 in the supplemental material. The oligonucleotides used in this work were synthesized by STAB Vida and are listed in Table S2. Restriction enzymes, T4 DNA ligase, and Phusion DNA polymerase were purchased from Fermentas. All enzymes were used according to the supplier’s instructions. *E. coli* strain DH5α was used for cloning experiments. pRMA03 was constructed by inserting a PCR-amplified DNA fragment carrying *bolA* promoter sequences (using RNM168 and RNM169 primers) into SalI and SmaI sites of vector pSP417. The resulting plasmid was transformed in MC1061 Δ*bolA* and MC1061 competent cells to obtain strains CMA812 and CMA813, respectively. The λRed-mediated mutagenesis method (46) was used to obtain the single *ydaM* (CMA815) and *yhjH* (CMA817) deletion strains. Briefly, for the Δ*ydaM* and Δ*yhjH* strains, a PCR fragment was obtained by amplification of pKD3 plasmid using primer pairs RNM236/237 and yhjHKOFor/yhjHKORev, respectively. The resulting fragments were transformed in MG1655 competent cells to allow recombination with the bacterial chromosome. P1 transduction was used to obtain strains CMA814, CMA816, and CMA818. CMA819 was constructed by transforming strain CMA816 with pCDA02 plasmid.

10.1128/mBio.00443-17.3 TABLE S1  Strains and plasmids used in this work. Download TABLE S1, DOCX file, 0.02 MB. Copyright © 2017 Moreira et al. 2017 Moreira et al. This content is distributed under the terms of the Creative Commons Attribution 4.0 International license.

10.1128/mBio.00443-17.4 TABLE S2  Oligonucleotides used in this work. Download TABLE S2, DOCX file, 0.1 MB. Copyright © 2017 Moreira et al. 2017 Moreira et al. This content is distributed under the terms of the Creative Commons Attribution 4.0 International license.

All constructs were confirmed by DNA sequencing at STAB Vida, Portugal.

### Bacterial growth conditions.

*E. coli* strains were grown in Luria-Bertani broth (LB) at 37°C with agitation, unless differently specified. When appropriate, antibiotics were used at the following concentrations: 100 µg/ml ampicillin, 50 µg/ml kanamycin, and 50 µg/ml chloramphenicol.

### Overexpression and purification of BolA protein.

BolA overexpression was performed using the pPFA02 plasmid and protein purification as previously described (39). The plasmid used for expression of BolA was the pET28a-derived pPFA02 plasmid (38), which was transformed into a Novagen *E. coli* BL21(DE3) strain. Purification of BolA was performed by histidine affinity chromatography using HisTrap chelating HP columns (GE Healthcare) and an AKTA fast protein liquid chromatography system (GE Healthcare).

### Motility assays.

Motility assays were performed as previously described (12). When appropriate, the plates were supplemented with 10 μM c-di-GMP (BioLog), pGpG (BioLog), guanosine (Sigma), cGMP (Sigma), GTP (Sigma), and cyclic AMP (cAMP; Sigma). Pictures were taken using ImageScanner III (GE Healthcare Life Sciences).

### c-di-GMP quantification.

Extraction and quantification of c-di-GMP were performed using bacterial liquid cultures obtained in the exponential and stationary phases, according to the method of Spangler et al. (47). The protein content of each bacterial culture was determined for normalization of c-di-GMP intracellular levels.

### Biofilm assays.

Crystal violet biofilm assays were performed as previously described by Dressaire et al. (12) with the following modifications. *E. coli* strains grown overnight in LB and diluted to a final optical density at 600 nm (OD_600_) of 0.1 in fresh LB media and incubated at 37°C. Once the early stationary phase was reached, the cultures were diluted to a final OD_600_ of 0.1 in M63 media supplemented with 0.2% of arabinose. Aliquots (200 µl) were transferred to a 96-well polyvinyl chloride (PVC) plate and incubated at 37°C without agitation for 24 h. The planktonic state was determined by measuring the OD_600_ of the unattached cells using a SpectraMax Plus 384 microplate reader (Molecular Devices). PVC plates were washed twice with Milli-Q (MQ) water and the attached bacteria stained with 0.1% crystal violet for 10 min. The crystal violet was solubilized by a solution of acetone-ethanol (1:4) and biofilm thickness estimated by measuring the OD_570_.

### Statistical analysis of differentially regulated genes.

Differentially regulated genes were identified as previously described (12). Analysis of Bayes statistics was used to evaluate the data, and the multiple-testing issue was taken into account through the calculation of the false-discovery rate (FDR) (48). Genes displaying a ratio associated with an FDR lower than 10% were considered differentially regulated.

### RNA extraction.

Total RNA extraction was performed as previously described (2). For qRT-PCR experiments, RNA samples were collected from cultures in the exponential and stationary phases. The extraction for RT-PCR was performed using bacterial cultures collected 3 h after BolA induction with 0.15% arabinose.

### Reverse transcription-PCR (RT-PCR).

RT-PCR reactions were performed as previously described (7). Primer pairs RNM080/RNM081, SB003/SB004, RNM082/RNM083, SB007/SB008, and RNM090/RNM091 were used to analyze *ydeH*, *yahA*, *yhjH*, *yhjK*, and *ydaM* expression, respectively. As an independent control, 16S rRNA was amplified with specific primers 16srrnF/16srrnR.

### Quantitative real-time PCR (qRT-PCR).

The expression of *bolA* mRNA was determined with a Rotor-Gene 3000 system (Corbett), using a SensiFast SYBR kit (Bioline) according to the supplier’s instructions. When necessary, bacterial cultures were supplemented with 1 μM c-di-GMP. qRT-PCR was performed with *bolA* gene-specific primers (RNM115 and RNM116). The *cysG* housekeeping gene was used as a control (RNM125 and RNM126). Relative quantifications of gene expression were calculated by the threshold cycle (ΔΔ*C*_*T*_) method (49).

### Determination of equilibrium dissociation constant (*K*_*D*_).

The *K*_*D*_ of the interaction of BolA with the substrates was determined using a BLItz system (ForteBio Inc.) according to the instructions of the manufacturer. Biotin-labeled DNA fragments were obtained by PCR, using RNM164*/RNM195, RNM196/RNM197*, RNM198*/RNM199, and RNM200*/RNM201 primers to amplify *ydaM*, *yhjH*, *yhjK*, and *ydeH* promoter regions, respectively. A negative-control experiment was performed with bovine serum albumin (BSA) protein and *flhDC* promoter sequence.

### β-Galactosidase activity assays.

The β-galactosidase activity of pRMA03 transcriptional fusion (Table S1) was assayed as described by Miller (50), with some modifications (12). The levels of expression of LacZ fusions were measured at specific time points during the entire growth cycle of bacteria. β-Galactosidase activity levels were expressed in Miller units using the following equation: Miller units = 1,000 × [OD_420_ − (1.75 × OD_550_)]/(*t* × *v* × OD_600_) (where *t* represents time and *v* represents volume).

### Accession number(s).

Microarray raw data (GenBank accession number GSE58509) were accessed from the Gene Expression Omnibus (GEO) (12).
