# Supplementary figures and images for: BolA Is Required for the Accurate Regulation of c-di-GMP, a Central Player in Biofilm Formation
Source: mBio. 2017 Sep 19;8(5):e00443-17. doi: 10.1128/mBio.00443-17 (PMC5605933; doi:10.1128/mBio.00443-17)

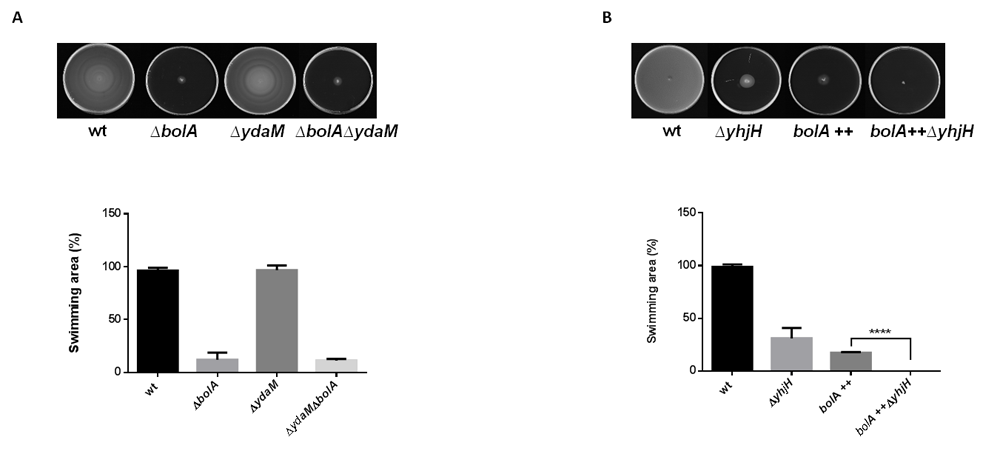

Supplement: FIG S1 [file mbo004173489sf1.tif]

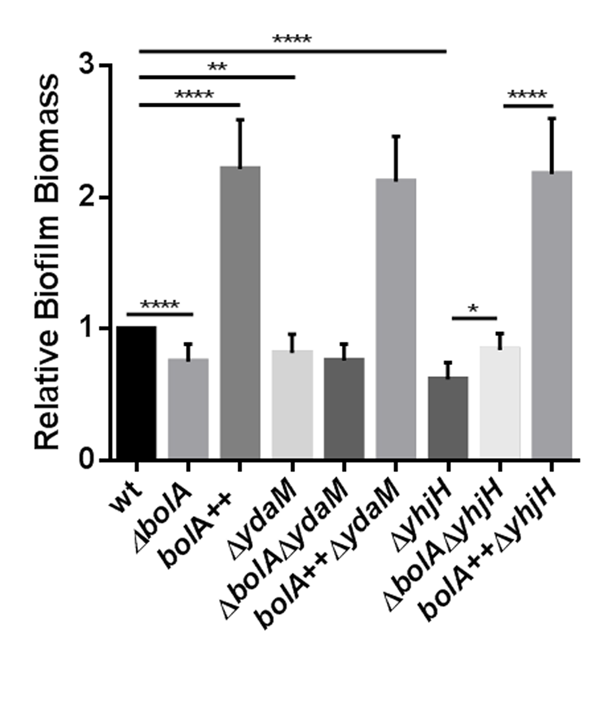

Supplement: FIG S2 [file mbo004173489sf2.tif]
