# Supplementary material for: BolA Is Required for the Accurate Regulation of c-di-GMP, a Central Player in Biofilm Formation
Source: mBio. 2017 Sep 19;8(5):e00443-17. doi: 10.1128/mBio.00443-17 (PMC5605933; doi:10.1128/mBio.00443-17)
Supplement: TABLE S1 [file mbo004173489st1.docx]

**Table S1** – Strains and plasmids used in this work

| Strain | Relevant marker (s) or genotype | Source or reference |  |
| --- | --- | --- | --- |
| **wt (CGSC 6300)** | MG1655 F^-^ λ^-^ rph-1 | ([1](#_ENREF_1)) |  |
| **MC1061** | F^-^ Δ(*ara-leu*)7697 [*araD*139]B/r Δ(*codB-lacI*)3 *galK*16 *galE*15 λ^-^ e14- *mcrA*0 *relA*1 *rpsL*150(*strR*) *spoT*1 *mcrB*1 *hsdR*2(r-m+) | ([2](#_ENREF_2)) |  |
| **DH5α** | *recA*1 *endA*1 *gyrA*96 *thi-hsdR*17 *supE*44 *relA*1 Δ(*lacZYA*^-^ *argF*)U169 ɸ80*dlacZ*ΔM15 | New England Biolabs |  |
| **BL21(DE3)** | F^–^ *ompT* *hsdSB*(rB^–^, mB^–^) *gal dcm* (DE3) | Invitrogen |  |
| **CMA50** | BL21(DE3) carrying pPFA02 plasmid | ([3](#_ENREF_3)) |  |
| **CMA94** | MG1655 Δ*bolA*::Kan^r^ | ([4](#_ENREF_4)) |  |
| **CMA95** | MG1655 Δ*bolA*::Kan^r^ carrying pCDA02 plasmid | ([4](#_ENREF_4)) |  |
| **CMA97** | MG1655 carrying pMAK580 plasmid | ([4](#_ENREF_4)) |  |
| **CMA98** | MG1655 Δ*bolA*::Kan^r^ carrying pBAD24 plasmid | ([4](#_ENREF_4)) |  |
| **CMA805** | MC1061 Δ*bolA*::Kan^r^ | ([4](#_ENREF_4)) |  |
| **CMA806** | MC1061 carrying pSP417 plasmid | ([4](#_ENREF_4)) |  |
| **CMA807** | MC1061 Δ*bolA*::Kan^r^ carrying pSP417 plasmid | ([4](#_ENREF_4)) |  |
| **CMA812** | MC1061 Δ*bolA*::Kan^r^ carrying pRMA03 plasmid | This study |  |
| **CMA813** | MC1061 carrying pRMA03 plasmid | This study |  |
| **CMA814** | MG1655 Δ*bolA*::Kan^r^ Δ*ycgR*::Cat^r^ | This study |  |
| **CMA815** | MG1655 Δ*ydaM*::Cat^r^ | This study |  |
| **CMA816** | MG1655 Δ*ydaM*::Cat^r^ Δ*bolA*::Kan^r^ | This study |  |
| **CMA817** | MG1655 Δ*yhjH*::Cat^r^ | This study |  |
| **CMA818** | MG1655 Δy*hjH*::Cat^r^ Δ*bolA*::Kan^r^ | This study |  |
| **CMA819** | MG1655 Δy*hjH*::Cat^r^ Δ*bolA*::Kan^r^ carrying pCDA02 plasmid | This study |  |
| **AB434** | MG1655 Δ*ycgR*::Frt | ([5](#_ENREF_5)) |  |
|  |  |  |  |
| Plasmid | Description | Origin/marker | Source or reference |
| **pPFA02** | pET28a plasmid encoding (His)_6_-BolA | Kan^r^ | ([3](#_ENREF_3)) |
| **pBAD24** | Arabinose-inducible plasmid | Amp^r^ | Invitrogen |
| **pMAK580** | pBR325 encoding *bolA* with its own promoters | pBR325/Cat^r^ | ([6](#_ENREF_6)) |
| **pCDA02** | pBAD encoding *bolA* | pBAD/Amp^r^ | ([4](#_ENREF_4)) |
| **pSP417** | Plasmid encoding *lacZ* | pBR322/Amp^r^ | ([7](#_ENREF_7)) |
| **pRMA03** | pSP417 encoding *lacZ* under the control of *bolA* promoters | pSP417/Amp^r^ | This study |
| **pKD3** | Plasmid encoding frt-flanked cat cassette | pANTSγ/Amp^r^;Cat^r^ | ([8](#_ENREF_8)) |

## References

1. **Guyer MS, Reed RR, Steitz JA, Low KB.** 1981. Identification of a sex-factor-affinity site in E. coli as gamma delta. Cold Spring Harb Symp Quant Biol **45 Pt 1:**135-140.

2. **Casadaban MJ, Cohen SN.** 1980. Analysis of gene control signals by DNA fusion and cloning in Escherichia coli. J Mol Biol **138:**179-207.

3. **Freire P, Moreira RN, Arraiano CM.** 2009. BolA inhibits cell elongation and regulates MreB expression levels. J Mol Biol **385:**1345-1351.

4. **Dressaire C, Moreira RN, Barahona S, Alves de Matos AP, Arraiano CM.** 2015. BolA is a transcriptional switch that turns off motility and turns on biofilm development. mBio **in press**.

5. **Boehm A, Kaiser M, Li H, Spangler C, Kasper CA, Ackermann M, Kaever V, Sourjik V, Roth V, Jenal U.** 2010. Second messenger-mediated adjustment of bacterial swimming velocity. Cell **141:**107-116.

6. **Aldea M, Hernandez-Chico C, de la Campa AG, Kushner SR, Vicente M.** 1988. Identification, cloning, and expression of bolA, an ftsZ-dependent morphogene of Escherichia coli. J Bacteriol **170:**5169-5176.

7. **Podkovyrov SM, Larson TJ.** 1995. A new vector-host system for construction of lacZ transcriptional fusions where only low-level gene expression is desirable. Gene **156:**151-152.

8. **Datsenko KA, Wanner BL.** 2000. One-step inactivation of chromosomal genes in Escherichia coli K-12 using PCR products. Proc Natl Acad Sci U S A **97:**6640-6645.
