# Supplementary material for: BolA Is Required for the Accurate Regulation of c-di-GMP, a Central Player in Biofilm Formation
Source: mBio. 2017 Sep 19;8(5):e00443-17. doi: 10.1128/mBio.00443-17 (PMC5605933; doi:10.1128/mBio.00443-17)
Supplement: TABLE S2 [file mbo004173489st2.docx]

**Table S2** – Oligonucleotides used in this work

| Oligo Name | Sequence 5'-3' |
| --- | --- |
| RNM019 | CATTATTCCCACCCAGAATAACC |
| RNM080 | CATTTCGACGCCTTTCAGGAGGG |
| RNM081 | CTGCACGACATGCTTCTTCATC |
| RNM082 | AGCCATCGGATGGAGGTTGTG |
| RNM083 | TAACGCACTTCACTTAGCGCAGAG |
| RNM090 | GAACTGCCGAAATACCTCTCC |
| RNM091 | AATCCTGAGCGATGGCGAGATGCTG |
| RNM115 | GGGTGAACGTTTTCTGAATCG |
| RNM116 | CAACCCTTCCCACTCCTTAATAG |
| RNM125 | GACGCTGGTGTTCTATATGGG |
| RNM126 | GGCATTCCGTGTTCAATCAG |
| RNM164* | AGTCCAGGGTATTGAAGTTGTG |
| RNM168 | ACGCGTCGACATGTTCGGGCTCTCAGAGAC |
| RNM169 | TCCCCCGGGGGAGAAATATCTCCTCCGACAAC |
| RNM188* | CGTGTAGTGACGAGTACAGTTG |
| RNM195 | CATCTCATCACCCCATTACC |
| RNM196 | CAAGCTCTCGATGCTTGCTTC |
| RNM197 | ACGCGCCGATAATCTTTGTC |
| RNM198* | GCCGTGTTATAGCGATTCTG |
| RNM199 | TTAATGCCGCTGCGATCGGG |
| RNM200* | TGGTAGTGGGCATCGATAGC |
| RNM201 | ACTGGGCCATTAGGGTATTGGG |
| RNM236 | CGATACAAAGCATCATCAACGCGTTTAAACAGTTCATCGAGTGTAGGCTGGAGCTGCTTC |
| RNM237 | CGTTTCGCCCTTTGAGGAACAGTTAATTTATGCCAATAGCCATATGAATATCCTCCTTAG |
| SB003 | CACGCCTGAAGCCATCAGTC |
| SB004 | AACCCGCCGAGACGTTGATG |
| SB007 | TTAACGCCATCCCAGCCAAG |
| SB008 | CCATCAGCAAGGCTTTGTTC |
| yhjHKOFor | AATCTTTGTCGAGTCCGGGCAGCATCACTTTTAAACACAGGACATCTTTGGTGTAGGCTGGAGCTGCTTC |
| yhjHKORev | TTCCTGTGCCAGTCCTAAAGATAGTCCAGCCAGGCGGAAAATGAGGCAGCCATATGAATATCCTCCTTAG |
| 16srrnF | GCTACAATGGCGCATACAAA |
| 16srrnR | TTCATGGAGTCGAGTTGCAG |

The * represents the biotin labeled oligonucleotides.
